# The usability of ventilators: a comparative evaluation of use safety and user experience

**DOI:** 10.1186/s13054-016-1431-1

**Published:** 2016-08-20

**Authors:** Plinio P. Morita, Peter B. Weinstein, Christopher J. Flewwelling, Carleene A. Bañez, Tabitha A. Chiu, Mario Iannuzzi, Aastha H. Patel, Ashleigh P. Shier, Joseph A. Cafazzo

**Affiliations:** 1 Healthcare Human Factors, Techna Institute, University Health Network, Toronto, Canada; 2 Institute of Health Policy, Management and Evaluation, University of Toronto, Toronto, Canada; 3 Institute of Biomaterials and Biomedical Engineering, University of Toronto, Toronto, Canada

**Keywords:** Comparative study, Human factors and ergonomics, Critical care, Patient safety, Ventilator, Mechanical, Medical device design

## Abstract

**Background:**

The design complexity of critical care ventilators (CCVs) can lead to use errors and patient harm. In this study, we present the results of a comparison of four CCVs from market leaders, using a rigorous methodology for the evaluation of use safety and user experience of medical devices.

**Methods:**

We carried out a comparative usability study of four CCVs: Hamilton G5, Puritan Bennett 980, Maquet SERVO-U, and Dräger Evita V500. Forty-eight critical care respiratory therapists participated in this fully counterbalanced, repeated measures study. Participants completed seven clinical scenarios composed of 16 tasks on each ventilator.

Use safety was measured by percentage of tasks with use errors or close calls (UE/CCs). User experience was measured by system usability and workload metrics, using the Post-Study System Usability Questionnaire (PSSUQ) and the National Aeronautics and Space Administration Task Load Index (NASA-TLX).

**Results:**

Nine of 18 post hoc contrasts between pairs of ventilators were significant after Bonferroni correction, with effect sizes between 0.4 and 1.09 (Cohen’s *d*). There were significantly fewer UE/CCs with SERVO-U when compared to G5 (*p* = 0.044) and V500 (*p* = 0.020). Participants reported higher system usability for G5 when compared to PB980 (*p* = 0.035) and higher system usability for SERVO-U when compared to G5 (*p* < 0.001), PB980 (*p* < 0.001), and V500 (*p* < 0.001). Participants reported lower workload for G5 when compared to PB980 (*p* < 0.001) and lower workload for SERVO-U when compared to PB980 (*p* < 0.001) and V500 (*p* < 0.001). G5 scored better on two of nine possible comparisons; SERVO-U scored better on seven of nine possible comparisons. Aspects influencing participants’ performance and perception include the low sensitivity of G5’s touchscreen and the positive effect from the quality of SERVO-U’s user interface design.

**Conclusions:**

This study provides empirical evidence of how four ventilators from market leaders compare and highlights the importance of medical technology design. Within the boundaries of this study, we can infer that SERVO-U demonstrated the highest levels of use safety and user experience, followed by G5. Based on qualitative data, differences in outcomes could be explained by interaction design, quality of hardware components used in manufacturing, and influence of consumer product technology on users’ expectations.

**Electronic supplementary material:**

The online version of this article (doi:10.1186/s13054-016-1431-1) contains supplementary material, which is available to authorized users.

## Background

Ventilators are a fundamental technology in critical care, with their use expected to increase in demand in the next 10 years [1]. Existing estimates of the proportion of patients admitted to the intensive care unit (ICU) requiring ventilator support range from 19 to 75 % in various countries [2–5]. The use of ventilators is not without risk to the patient, with potential harm arising from infections, pneumothorax, ventilator-associated lung injury, and oxygen toxicity [6–9].

Other significant ventilator-related risks are the associated use errors with the device [10–14]. Use errors could cause patient harm in their operation if devices are not properly designed to mitigate such risks [11, 15, 16]. The design of ventilators can negatively affect user performance through poor user interfaces, interaction modes, or difficulties during the physical setup of the equipment [17].

The evaluation of use safety and the user experience of medical devices can be conducted through usability testing [18, 19]. Usability testing of medical devices has become increasingly important in recent years, with the US Food and Drug Administration (FDA) requiring medical devices to satisfy minimum use safety requirements prior to regulatory approval [20]. However, the testing conducted by manufacturers for their FDA submissions is often confidential, qualitative in nature, and not intended to achieve statistical significance in validating the product design [18]. As such, there are no means to compare the outcomes of these studies with those of similar devices on the market or with findings from other studies in the literature [21].

To address this limitation, a comparative usability test can be used, where multiple devices are evaluated concurrently, following the same protocol [18, 22]. Existing comparative studies available in the literature, however, normally test two devices or variations of a device design, as with, for example, the testing of compact transport ventilators [23], laparoscopic devices [24], and inhalers [25]. Other studies that provide comparisons of a larger number of devices rely on simplified methodologies that lack scientific rigor [26, 27].

In order to compare the use safety and user experience of critical care ventilators on the market, it was necessary to design and run a comparative usability test with a large enough sample of representative users to determine if there were statistically significant differences among ventilators.

This study’s intent is to provide empirical evidence of the difference in use safety and user experience of four market-leading critical care ventilators available in North America [28]: the Hamilton G5 (Hamilton Medical AG; Bonaduz, Switzerland), the Covidien Puritan Bennett 980 (Covidien LP; Mansfield, MA, USA), the Maquet SERVO-U (Maquet Critical Care AB; Solna, Sweden), and the Dräger Evita Infinity V500 (Dräger Medical GmbH; Lübeck, Germany). The findings explore the design of the ventilators in two dimensions of interest: use safety and user experience (a combination of perceived system usability and workload). The methodology presented in this paper enables users and decision makers to better understand the differences between designs of mechanical ventilators on the market, thereby supporting an understanding of user needs and procurement processes alike [29].

## Methods

### Experimental design

In order to estimate the sample size and the feasibility of a full-scale, powered study [30–32], it was necessary to obtain a priori knowledge regarding the performance of the selected ventilators. As this data was not available in the literature, a pilot study with 13 participants was performed. Sample size was calculated assuming a repeated measures design with possible contrasts, with 80 % power and significance at the 5 % level [33] for use safety, system usability, and workload. Pilot data indicated a minimum of 48 participants was needed to discriminate between how each of the ventilators performed using the three selected metrics. This sample size allowed a full counterbalancing of the order of ventilators and the use of a repeated measures design to account for learning effects, order effects, and fatigue [30–32]. Data analyses for the pilot study and the full-scale study were performed by the principal investigator, who was blinded to which dataset corresponded to which ventilator, to ensure objectivity.

The study was conducted at the Clinical Skills and Patient Simulation Center (CSPSC) at the University of North Carolina School of Medicine (see Additional file 1) and received ethics approval (HHF1520_3) by the Quorum Review Independent Review Board (Quorum Review, Seattle, WA, USA). Written consent was collected from all study participants for the publication of their de-identified data and accompanying images used in this manuscript.

### Participants

A total of 13 respiratory therapists (RTs) participated in the pilot study and 48 RTs in the full-scale study. RTs were chosen as the target group for this study because they are the primary daily users of ventilators in North American hospitals. The RT in the United States is responsible for “*clinical decision-making and patient education, [the RT] develops and implements respiratory care plans, applies patient-driven protocols, utilizes evidence-based clinical practice guidelines, and participates in health promotion, disease prevention, and disease management*.” [34]. RTs are responsible for responding in emergency situations, initiating and managing ventilators, and providing airway management in high-risk areas of North American hospitals, such as ICUs and emergency departments [34, 35].

Participants for this study were recruited from three hospital networks in North Carolina (Duke Health, WakeMed Health & Hospitals, and UNC Health Care). The CSPSC helped with recruitment by forwarding the recruitment email to head RTs in the three hospital networks, and a total of 143 RTs responded to the recruitment email. A survey was used during recruitment for selecting a group of participants with experience balanced among the four families of ventilators, to identify RTs with experience in critical care and to avoid recruiting participants with consulting relationships with manufacturers (see Additional file 2).

### Devices

Each participant performed the scenarios on all four ventilators: the Hamilton G5 (G5), the Covidien Puritan Bennett 980 (PB980), the Maquet SERVO-U (SERVO-U), and the Dräger Evita Infinity V500 (V500). The order of ventilators was fully counterbalanced, to avoid learning and order effects, and a repeated measures study design was used. These ventilators were selected for the study as the most advanced models from the North American market leaders [28].

### Tasks and scenarios

The internationally recognized standard, ISO 80601-2-12 — Particular requirements for basic safety and essential performance of critical care ventilators, details the primary operating functions in a ventilator-neutral and independent manner [36]. The 16 representative tasks described in Table 1 were determined based on the aforementioned standard.

**Table 1:** Description of scenarios and associated tasks, as well as alarms and alerts

Scenario	Tasks
1	Urgent ventilator parameter setup and start ventilation^a^, adjust alarm limits
2	Activate expiratory pause, inspiratory pause
3	Read respiratory rate from a distance, adjust respiratory rate, view data not available in default view, and suction
4	Leak test
5	Wean from pressure control to synchronized intermittent mandatory ventilation and adjust trigger
6	Return to previous mode
7	Standby
Alarms/alerts	Loss of oxygen^a^ and power failure^a^

^a^Tasks where delayed action could potentially lead to harm to the patient

A total of seven scenarios were developed to create relevant clinical context, incorporating both typical clinical scenarios and time-sensitive scenarios, such as response to a loss of oxygen supply. The scenarios were designed by the authors and later vetted through consultations with RTs, ensuring accurate reflection of clinical context and patient conditions. The scenarios and tasks were always presented in the same order to maintain the clinical context, with the exception of the alarm tasks, which were randomized. All tasks had a 10-minute time limit, with a 160-minute time limit per ventilator.

In order to address concerns over instructor-led manufacturer training [37], this study employed exploration-based training [38] to increase realism and alignment with the delivery of training in real conditions (which was confirmed during the pilot study). Participants were given a set of learning objectives and asked to familiarize themselves with each of the four ventilators until they felt they would be comfortable using them on a real patient. A test administrator was available to answer questions and demonstrate any functions. At a minimum, all participants were required to demonstrate the ability to independently ventilate the patient, adjust parameters, adjust alarm limits, and browse menus.

### Variables

Use safety, system usability, and workload were selected as the three major measures of interest, since they correlate to aspects of a medical device’s design quality and are known to affect patient safety [20, 39–42]. These variables were measured through a combination of observed use and validated subjective scales, which measured participants’ perception of the four devices and provided a comprehensive view of both the RTs’ perception and actual performance when using the ventilators. A combination of observed use and self-reported perception was used to protect this study from bias that may relate to the “preference versus performance paradox” [43, 44].

Use safety was measured as inversely proportional to the percentage of tasks (total of 16) in which participants had a use error or close call (UE/CC); hence, a lower percentage represents a safer device. UE/CCs were collected through well-established observation techniques [18–20]. A use error is defined as an action (or failure to act) that directly compromises safety or effectiveness of a device or that results in an undesirable or unintended treatment. A close call is defined as an instance in which a user experiences a usability issue that would result in a use error but successfully recovers prior to compromising the task. Two human factors experts independently observed each participant completing the tasks and recorded whether a UE/CC occurred during a task. This variable was categorical (UE/CC or no UE/CC), so a clinical task was completed either with a UE/CC or without one. Following data collection, observers compared ratings on a task-by-task basis and agreed on any clarifications to be sought during the participant debriefing. In cases where the issue remained unclear, a third human factors professional would independently resolve the tie through video review.

System usability was evaluated through a combination of the UE/CC metric described above and the Post-Study System Usability Questionnaire (PSSUQ) [45]: a 16-question, self-reported subjective evaluation of perceived system usability. The PSSUQ has been used in healthcare to evaluate clinical monitoring [46], anesthesia [47], as well as telerehabilitation systems [48]. PSSUQ scores range from 1 to 7, with lower scores representing better perceived system usability.

Lastly, workload was evaluated using the National Aeronautics and Space Administration Task Load Index (NASA-TLX) [49, 50], a subjective workload assessment tool that relies on six subscales (Mental Demand, Physical Demand, Temporal Demand, Own Performance, Effort, and Frustration). NASA-TLX has been used extensively in healthcare [50] to evaluate medical devices such as ventilators [51], infusion pumps [39], and physiological monitoring displays [52]. The output from the NASA-TLX instrument is a score ranging from 0 to 100, where lower scores correspond to lower perceived workload.

### Data collection

Each participant session lasted a maximum of 8 hours, which included a 1.5-hour exploratory training period followed by four 1.5-hour periods (with breaks between periods), where participants performed the tasks on each of the four ventilators. For each ventilator, participants went through the scenarios described in Table 1 while being observed by human factors experts responsible for logging the occurrence of UE/CCs and for collecting qualitative data about the performance of the participants. At the end of each sequence of scenarios, participants were presented with PSSUQ and NASA-TLX tools to evaluate the ventilator they had just used, followed by a debriefing interview to allow them to voice their opinions. This same process was repeated for each of the three remaining ventilators.

### Data analysis

Statistical analyses were performed using SPSS Version 22.0 (IBM Corp, Armonk, NY, USA). Overall differences in safety and user experience of the ventilators were explored through repeated measures analysis of variance (ANOVA). Post hoc contrasts comparing any two ventilators were performed using multiple pairwise *t* tests. [30, 31]. Bonferroni corrections were used due to the multiple comparisons performed, with other less conservative corrections having minor effects on the number of statistically significant comparisons. Bonferroni correction can be overly conservative in studies of this type, considering that the outcome variables are correlated, increasing the chance of false negatives [53]. For this reason, the results of contrasts with Bonferroni correction and the post hoc *t* tests without correction are both reported in the results section (see Table 4 for uncorrected results).

## Results

A summary indicating how each pair of ventilators compares is presented in Table 2, where only the statistically significant pair comparisons are presented. The SERVO-U outperformed the other ventilators in seven out of nine possible pair comparisons, and the G5 outperformed the other ventilators in two out of nine possible comparisons. The PB980 and the V500 did not outperform the other ventilators.

**Table 2:** Comparative description of how any two ventilators compare^a^

	Safer	Better perceived usability	Lower workload
	(Observed UE/CC)	(PSSUQ)	(NASA-TLX)
Hamilton G5 compared to Puritan Bennett PB980		G5	G5
Hamilton G5 compared to Maquet SERVO-U	SERVO-U	SERVO-U	
Hamilton G5 compared to Dräger V500			
Puritan Bennett PB980 compared to Maquet SERVO-U		SERVO-U	SERVO-U
Puritan Bennett PB980 compared to Dräger V500			
Maquet SERVO-U compared to Dräger V500	SERVO-U	SERVO-U	SERVO-U

*UE/CC* use error/close call, *PSSUQ* Post-Study System Usability Questionnaire, *NASA-TLX* National Aeronautics and Space Administration Task Load Index

^a^Only statistically significant results after Bonferroni corrections are presented

### Overall ventilator comparison

Table 3 outlines the percentage of tasks with UE/CCs, the perceived workload of each ventilator on the NASA-TLX scale, and the usability of the different ventilators as measured by the PSSUQ scale. Box plots, presented as an Additional file 3, provide a visual representation of these data.

**Table 3:** Ventilator performance in the UE/CC, NASA-TLX, and PSSUQ metrics

	Use error and close calls (% of tasks) Use safety		NASA-TLX (0–100) Workload		PSSUQ (1–7) System usability	
Ventilator	Mean	Standard deviation	Mean	Standard deviation	Mean	Standard deviation
G5	12.8	10.7	28.3	20.5	2.7	1.3
PB980	13.2	11.9	43.7	21.9	3.5	1.5
SERVO-U	9.1	11.0	21.5	17.1	1.7	0.9
V500	16.9	14.1	34.6	20.8	3.1	1.4

Lower scores on all three metrics correspond to better perception/performance

*UE/CC* use error/close call, *NASA-TLX* National Aeronautics and Space Administration Task Load Index, *PSSUQ* Post-Study System Usability Questionnaire

Repeated measures ANOVA showed statistically significant differences on all three variables: *UE/CC*, F(2.5, 119.1) = 6.101, *p* < 0.001, partial η^2^ = 0.115; *NASA-TLX*, F(3, 141) = 16.629, *p* < 0.001, partial η^2^ = 0.261; and *PSSUQ*, F(3, 141) = 17.821, *p* < 0.001, partial η^2^ = 0.275. Residuals were normally distributed.

### Ventilator pair comparison

Six post hoc comparisons with Bonferroni correction [30, 31, 53, 54] were performed for each metric, which allowed pairs of ventilators to be ranked in terms of use safety (UE/CC), system usability (PSSUQ and UE/CC), and workload (NASA-TLX). The contrasts look at the differences in the means (M_D_) for each metric and determine, after corrections, whether these differences are statistically significant (Table 4). After applying Bonferroni corrections, nine out of the 18 possible comparisons were statistically significant.

**Table 4:** Mean differences (M_D_ *=* Vent_1_ – Vent_2_) between the ventilators with the results of the post hoc contrasts with Bonferroni correction (df = 47), the post hoc *t* tests without corrections, and the effect sizes (Cohen’s *d*)

		Contrasts with Bonferroni correction		Post hoc *t* tests without correction		Effect size
		M_D_ (Mvent_1_ *−* Mvent_2_)	*p*	*t*(47)	*p*	Cohen’s *d*
Use error/close calls (%) Use safety						
Vent_1_	Vent_2_					
G5	PB980	−0.391	1.000	−0.209	0.835	0.03
G5	SERVO-U	3.646	0.044^a^	2.804	0.007^a^	0.40
G5	V500	−4.167	0.292	−2.024	0.049^a^	0.29
PB980	SERVO-U	4.036	0.149	2.319	0.025^a^	0.33
PB980	V500	−3.776	0.287	−2.032	0.048^a^	0.29
SERVO-U	V500	−7.813	0.002^a^	−3.824	<0.001^a^	0.55
NASA-TLX (0–100) Workload						
Vent_1_	Vent_2_					
G5	PB980	−15.449	< 0.001^a^	−4.404	< 0.001^a^	0.64
G5	SERVO-U	6.765	0.153	2.308	0.025^a^	0.33
G5	V500	−6.379	0.547	−1.725	0.091	0.25
PB980	SERVO-U	22.214	< 0.001^a^	7.524	< 0.001^a^	1.09
PB980	V500	9.070	0.072	2.615	0.012	0.38
SERVO-U	V500	−13.144	< 0.001^a^	−4.323	< 0.001^a^	0.62
PSSUQ (1–7) System usability						
Vent_1_	Vent_2_					
G5	PB980	−0.807	0.035^a^	−2.884	0.006	0.42
G5	SERVO-U	0.935	< 0.001^a^	4.363	< 0.001^a^	0.63
G5	V500	−0.452	0.508	−1.761	0.085	0.25
PB980	SERVO-U	1.742	< 0.001^a^	7.456	< 0.001^a^	1.07
PB980	V500	0.354	1.000	1.195	0.238	0.17
SERVO-U	V500	−1.388	< 0.001^a^	−6.221	< 0.001^a^	0.87

Negative *M*_*D*_ values representing Vent_1_ performing better than Vent_2_

^a^Statistically significant results

Participants experienced fewer UE/CCs with the SERVO-U (9.1 %) than with the G5 (12.8 %), M_D_ = −3.646, *p =* 0.044, *d =* 0.40*.* Participants also experienced fewer UE/CCs with the SERVO-U (9.1 %) than with the V500 (16.9 %), M_D_ = −7.813, *p =* 0.002, *d =* 0.55.

On the PSSUQ metric (ranging from 1 to 7), participants reported better usability for the G5 (2.7) than for the PB980 (3.5), M_D_ = −0.807, *p =* 0.035, *d =* 0.42. They also perceived better usability for the SERVO-U (1.7) compared to the G5 (2.7), PB980 (3.5), and V500 (3.1), M_D_ = −0.935, *p <* 0.001, *d =* 0.63; M_D_ = −1.742, *p <* 0.001, *d =* 1.07; M_D_ = −1.388, *p <* 0.001, *d =* 0.87, respectively.

Lastly, on the NASA-TLX metric (ranging from 0 to 100), participants reported lower workload for the G5 (28.3) compared to the PB980 (43.7), M_D_ = −15.449, *p <* 0.001, *d =* 0.64. They also reported lower workload for the SERVO-U (21.5) compared to the PB980 (43.7) and V500 (34.6), M_D_ = −22.214, *p <* 0.001, *d =* 1.09 and M_D_ = −13.144, *p <* 0.001, *d =* 0.62, respectively.

Effect sizes were within the 0.4 to 1.09 range, with most comparisons having medium (*d* > 0.5) and strong (*d* > 0.8) effect sizes (see Table 4 for the complete results) [33].

### Demographics

Data were collected from 48 participants for the full-scale study, out of which 34 % were male (n = 16) and 66 % were female (n = 32), with 68 % of the participants being between the ages of 25 and 45 years old (n = 33). As for experience, 63 % of the RTs who participated in the study (n = 30) had five or more years of experience as an RT.

A perfect balance of participants’ level of experience with each of the ventilators was not possible due to uneven market share of the ventilators. However, using the data collected through the recruitment survey, multiple regression models were performed for all variables collected in the study, showing only minor effects on PSSUQ scores for the PB980, F(4,43) = 4.796, *p* = 0.003, adj. R^2^ = 0.24, where only the experience with the PB980 (*p* = 0.044, β = −0.268) and the G5 (*p* = 0.034, β = −0.347) had an effect on the PSSUQ score for the PB980. All other variables collected in this study were not influenced by the experience with the ventilators.

## Discussion

The intent of this study was to provide empirical evidence of the differences in use safety and user experience of four market-leading critical care ventilators available in North America. As the scenarios were the same for all four ventilators, the results presented in this paper suggest that the different user interfaces and interaction designs, as well as the quality of the hardware used, may have had an impact on user performance and perception. Additionally, the results reinforce the importance of user interfaces and user interaction in the design of medical technology [55–57] as well as in the quality of the hardware used in manufacturing. For instance, the lack of sensitivity of the G5’s touchscreen proved to be a barrier for task completion and a significant source of frustration, while the SERVO-U’s user interface was praised by the participants. The design of a medical technology is a factor that can strongly influence user experience and user performance, as widely discussed in the medical device and critical care literature [55, 57–59]. These results are also of critical importance for patient safety as they serve as an indicator of which medical technology is less likely to produce adverse events [55, 60, 61] arising from the operation of the devices.

The four ventilators were compared using repeated measures ANOVA, and we found statistically significant differences on all three variables (NASA-TLX, PSSUQ, UE/CC), showcasing medium (partial η^2^ > 0.06) to large (partial η^2^ < 0.13) effect sizes [33]. These results validate the sensitivity of our study design to discriminate the performance of the ventilators.

The participants’ opinions were further supported by the results of the paired contrasts through repeated measures *t* test. The data from Table 2 show that SERVO-U outperformed other ventilators in seven out of nine comparisons with other ventilators, showing medium to large effect sizes. These results indicate that participants’ perceptions of the SERVO-U’s superior user interface were reflected in the subjective and objective data collected in the study. SERVO-U showed safer performance (measured through UE/CC) when compared to the G5 and the V500, better perceived usability when compared to any of the three other ventilators, and lower perceived workload when compared to the PB980 and the V500. Next, the Hamilton G5 outperformed the PB980, both in self-reported usability and workload. The PB980 and the V500 did not outperform any ventilator in this study. Within the scope of this project, the SERVO-U, followed by the G5, demonstrated the highest levels of use safety and user experience, both factors that can directly impact patient safety [20, 40–42].

Using only the quantitative results, it is not possible to ascertain which specific factors influenced user performance. Hence the importance of also collecting qualitative data in the form of observations to further enrich the analysis [18, 20]. The qualitative data also collected in this study indicate that the choices of interaction model of each ventilator (e.g., how to select information on the screen, adjusting settings, and confirming) seem to interfere with task completion and affect users’ overall perception of the devices. A more detailed description of operational difficulties and safety implications of design should be explored in future publications, promoting an in-depth assessment of problems observed in this study.

The method used provided a comprehensive view of user experience and use safety of ventilators. NASA-TLX [49, 50], PSSUQ [45], and UE/CC [18–20] have demonstrated their capacity for discriminating participants’ performance on the ventilators, as well as for ranking the performance of medical devices available on the market. Even after applying Bonferroni corrections [53], our methodology was still able to discriminate the ventilators in 50 % of the possible comparisons (9/18 cases). In Europe, the tasks completed by RTs in this study are normally performed by nurses and doctors. Future studies could potentially compare the performance of RTs in North America with that of nurses and physicians in Europe.

Ultimately, the goal of this methodology is to support the design and/or selection of the safest medical devices on the market. The FDA, as well as researchers in patient safety, all posit the strong relationship that medical device usability has with use and patient safety [20, 40–42], where devices with poor usability can potentially lead to harm to the patient. Hence, such a strong relationship should be reflected in our results. This effect was observed when comparing the SERVO-U with the V500 and G5 but not when comparing the SERVO-U to the PB980. This difference was a result of the conservative nature of Bonferroni corrections [53]. The uncorrected UE/CC comparison of the SERVO-U and PB980 is significant (see Table 4), further supporting the relationship already discussed in the literature between usability and use safety.

In terms of further exploring the safety of medical technology, several studies in critical care that primarily focus on general characteristics and technical performance of medical devices would benefit from the rigorous methodology presented in this paper, to afford the evaluation of the human component on the use of technology, for example, studies of point-of-care technology [62] or emergency and transport ventilators [63], as well as those evaluating the effectiveness of electronic physician order entry in the ICU [59]. The effect of the human component has been extensively discussed in the critical care literature [58, 64, 65], describing how the design of human–machine interfaces (or of medical device user interfaces) play an important role in the safety of critical care technology [56, 57].

Limitations of this study include the fidelity of simulated conditions and that only four ventilators were tested. Only RTs were included in the study, as opposed to nurses and physicians, who tend to be primary users outside North America. Additionally, the recruitment criteria and the structure of the demographic data limited our ability to run a regression analysis to evaluate the effect of different demographics variables on the variables being measured. Our study was not powered to run such regression analysis.

Lastly, this study was sponsored by the Maquet Getinge Group. Precautions and safeguards were taken to ensure the independence of the research. The study design, development of the methodology, selection of variables, data analysis, and manuscript preparation were made independently of the project sponsor. As we did not know how the ventilators would perform, a pilot study was used both for the calculation of sample size and to test the hypothesis that there would be measurable differences between ventilators. To further the independence of our research, all the statistical analyses were performed by the principal investigator, who was blinded to the identity of the ventilators.

## Conclusions

This study provides empirical evidence on how the four ventilators from market leaders compare and highlights the importance of the design of medical technology. Within the boundaries of this study, we can infer that the SERVO-U ventilator demonstrated the highest levels of use safety and user experience, followed by the G5. Based on qualitative data collected during this study, differences in outcomes could be explained by interaction design, quality of the hardware components used in manufacturing, and influence of consumer product technology on users’ expectations. Ultimately, the results presented in this paper provide evidence of the feasibility and potential of novel methodology comparative usability testing in identifying the safest and most usable medical technology on the market, supporting the selection of the safest medical technology and the design of the next generation of devices.

## Abbreviations

ANOVA, analysis of variance; CSPSC, Clinical Skills and Patient Simulation Center; FDA, Food and Drug Administration; ICU, intensive care unit; NASA-TLX, National Aeronautics and Space Administration Task Load Index; PSSUQ, Post-Study System Usability Questionnaire; RT, respiratory therapist; UE/CC, use error/close call

## Additional files

Additional file 1: Setup of the testing facilities at the Clinical Skills and Patient Simulation Center at the University of North Carolina School of Medicine, where the simulator room and observation room can be seen. (PDF 1830 kb)

Additional file 2: Recruitment survey used in this study. (PDF 641 kb)

Additional file 3: Box plots showing the performance of the four ventilators in the use error/close call metric, the NASA-TLX scale, and the PSSUQ scale. *Shading* is used simply to differentiate datasets. *Dots* represent outliers. Lower scores on all three metrics correspond to better perception/performance. (PDF 72.4 kb)
