# Supplementary figures and images for: The usability of ventilators: a comparative evaluation of use safety and user experience
Source: Crit Care. 2016 Aug 20;20:263. doi: 10.1186/s13054-016-1431-1 (PMC4992292; doi:10.1186/s13054-016-1431-1)

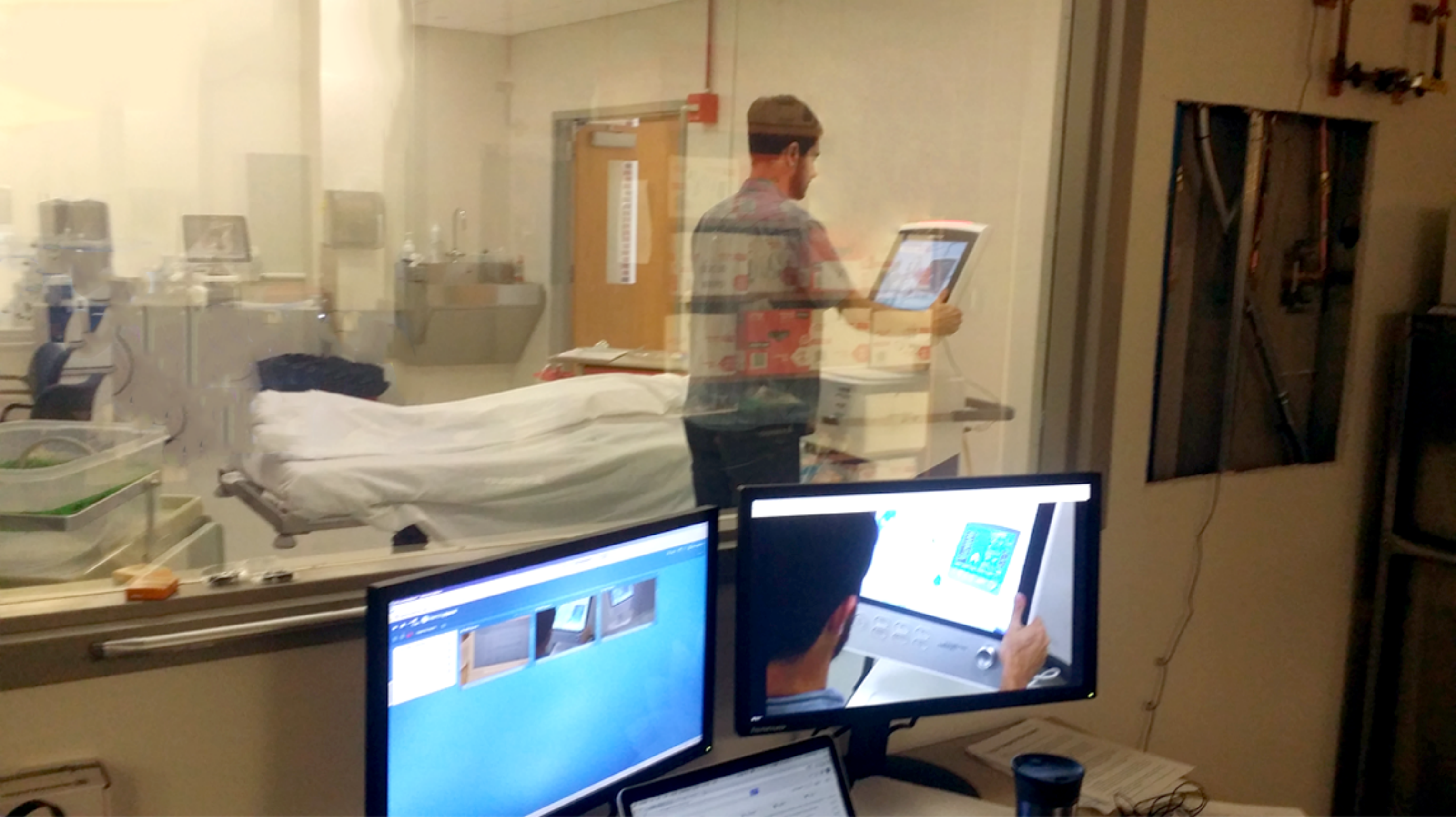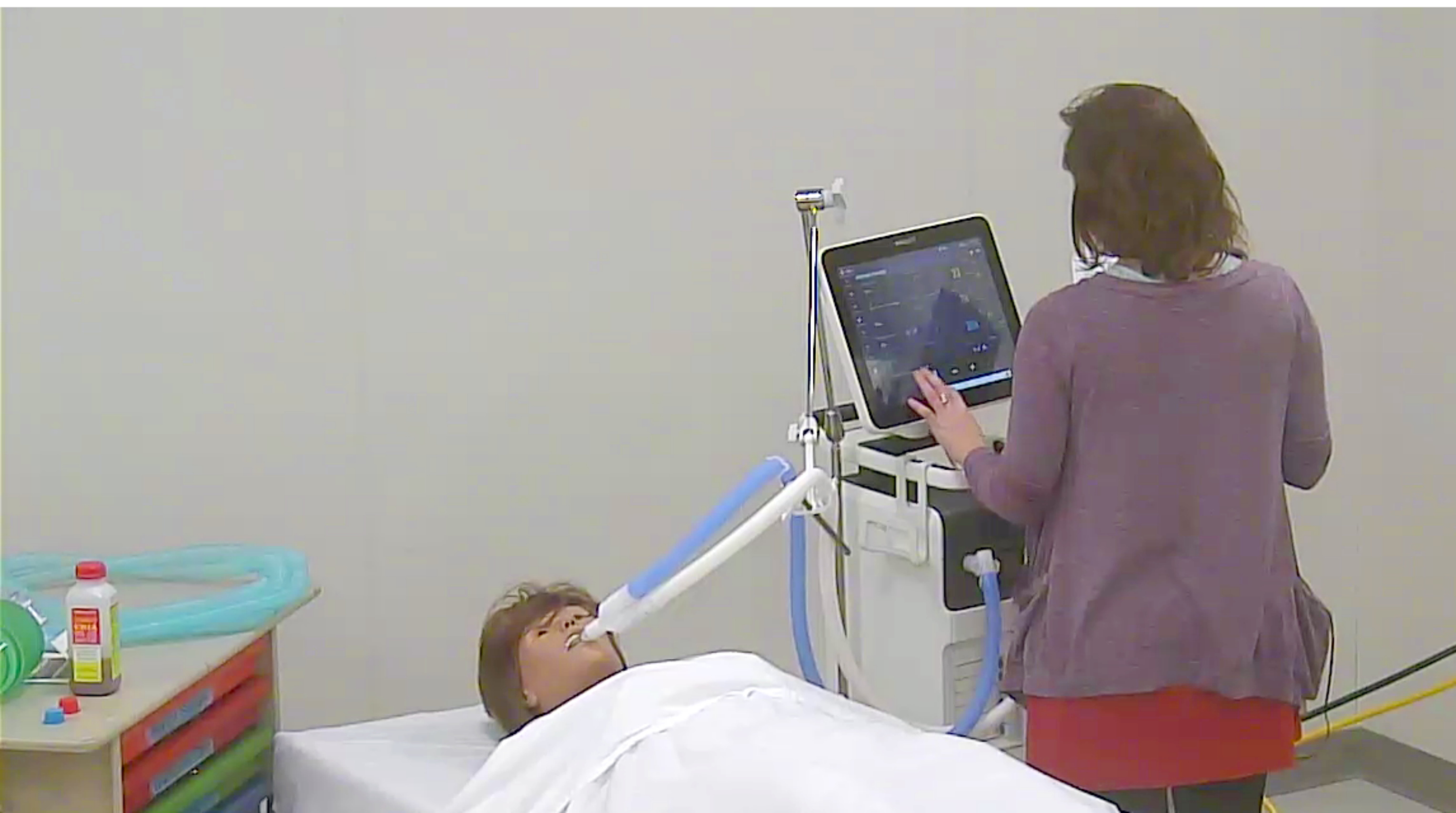

Supplement: Supplementary file 1 — Setup of the testing facilities at the Clinical Skills and Patient Simulation Center at the University of North Carolina School of Medicine, where the simulator room and observation room can be seen. (PDF 1830 kb) [file 13054_2016_1431_MOESM1_ESM.pdf]

## Use Errors/Close Calls

Use Safety

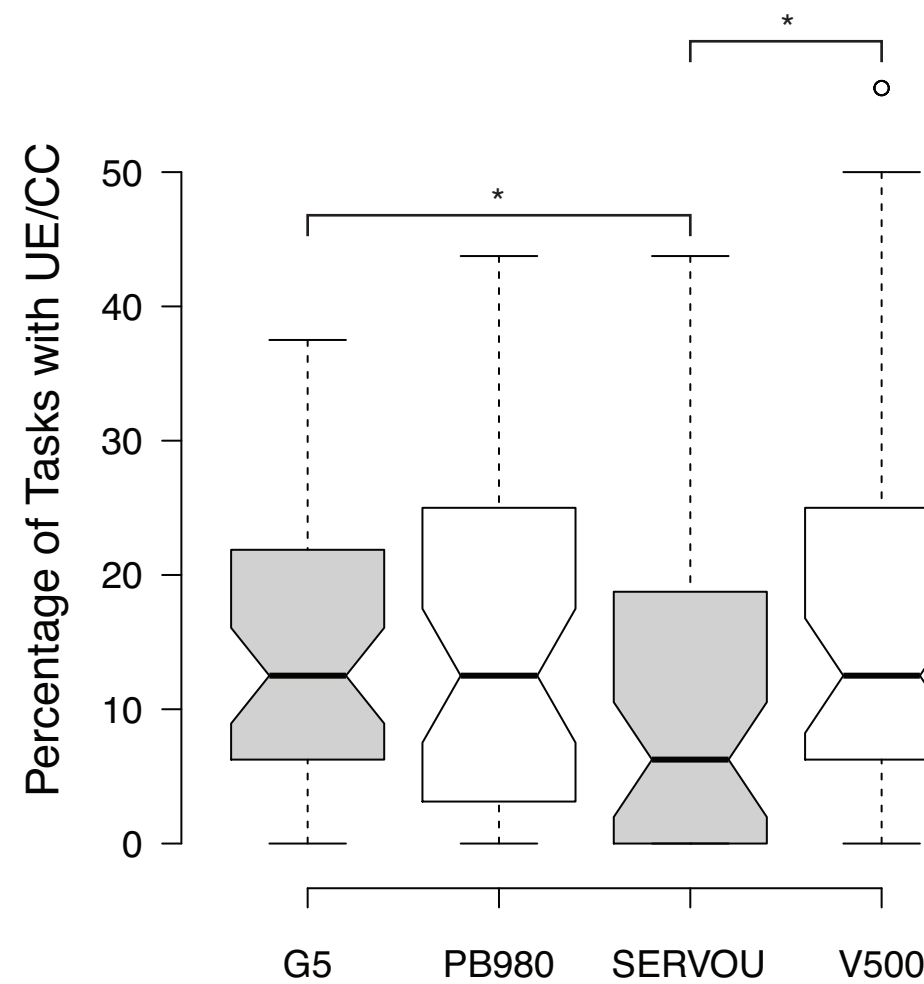

## NASA-TLX

Workload

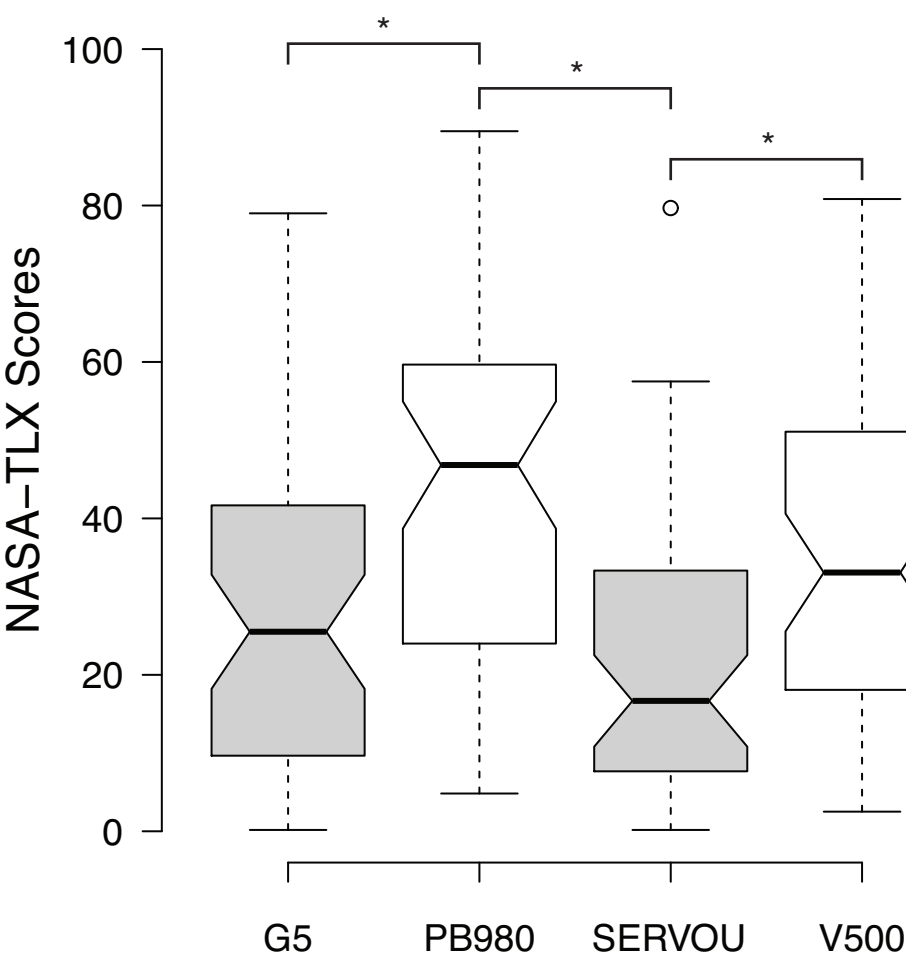

## PSSUQ

System Usability

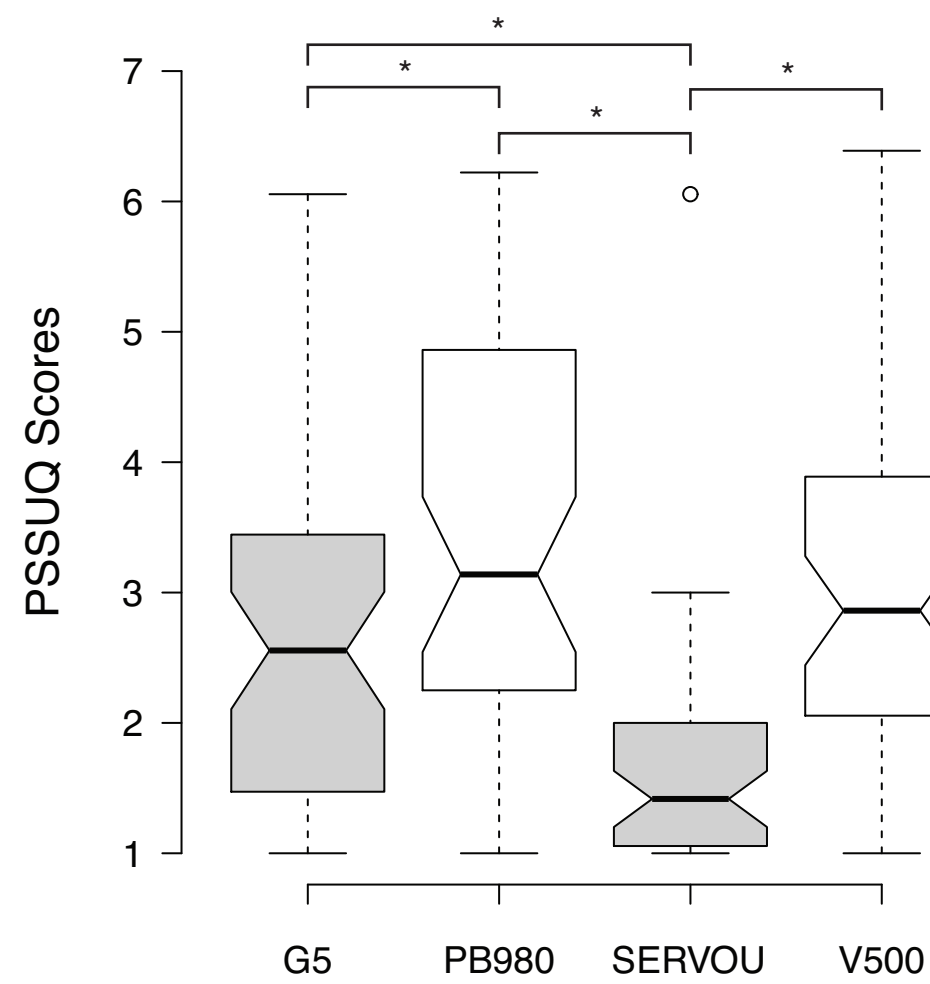

Supplement: Supplementary file 3 — Box plots showing the performance of the four ventilators in the use error/close call metric, the NASA-TLX scale, and the PSSUQ scale. Shading is used simply to differentiate datasets. Dots represent outliers. Lower scores on all three metrics correspond to better perception/performance. (PDF 72.4 kb) [file 13054_2016_1431_MOESM3_ESM.pdf]
