# Supplementary material for: The usability of ventilators: a comparative evaluation of use safety and user experience
Source: Crit Care. 2016 Aug 20;20:263. doi: 10.1186/s13054-016-1431-1 (PMC4992292; doi:10.1186/s13054-016-1431-1)
Supplement: Supplementary file 2 — Recruitment survey used in this study. (PDF 641 kb) [file 13054_2016_1431_MOESM2_ESM.pdf]

Healthcare Human Factors
University Health Network

Ventilator Comparison Evaluation

* 1. First Name

* 2. Last Name

* 3. Email address

* 4. Phone number

We recommend entering your mobile number.

* 5. How long have you been a Registered Respiratory Therapist?

- Less than 5 years
- 5-15 years
- 15 or more years

6. In what age range do you belong?

- <25 years
- 25-34 years
- 35-44 years
- 45-54 years
- 55-64 years
- >65 years

7. In which hospital(s) do you currently work?

* 8. Which patient population do you currently work with primarily?

- Adult
- Pediatric
- Neonatal

Other (please specify)

* 9. In which unit(s) of the hospital do you primarily work in?

- In-patient unit
- Emergency
- Intensive care unit

Other (please specify)

* 10. Within the last month, how often have you set up a ventilator and/or adjusted ventilator settings to assist with patient breathing?

- Never
- Once
- At least once a week
- Several times a week
- Every day
- Other (please specify)

* 11. Have you ever had a consulting relationship with a company that develops or sells ventilators?

- Yes
- No

If yes, please provide details:

Healthcare Human Factors
University Health Network

Ventilator Comparison Evaluation

The Puritan Bennett 840 and Puritan Bennett 980 are pictured below.

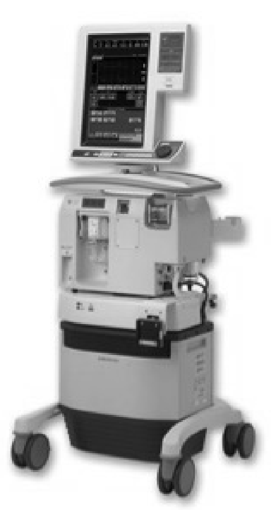

Puritan Bennett 840

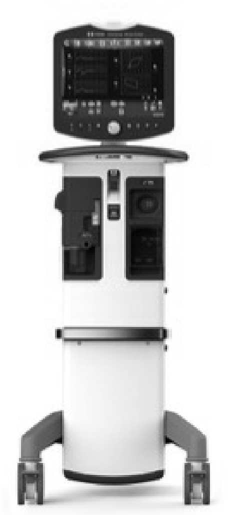

Puritan Bennett 980

The Hamilton S1 and Hamilton G5 are pictured below.

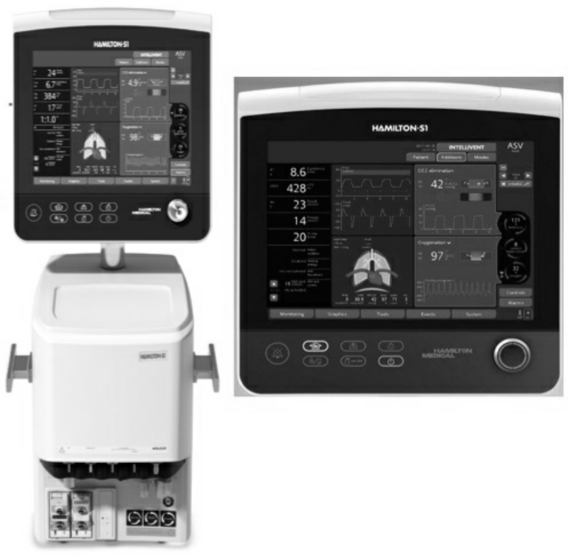

Hamilton S1

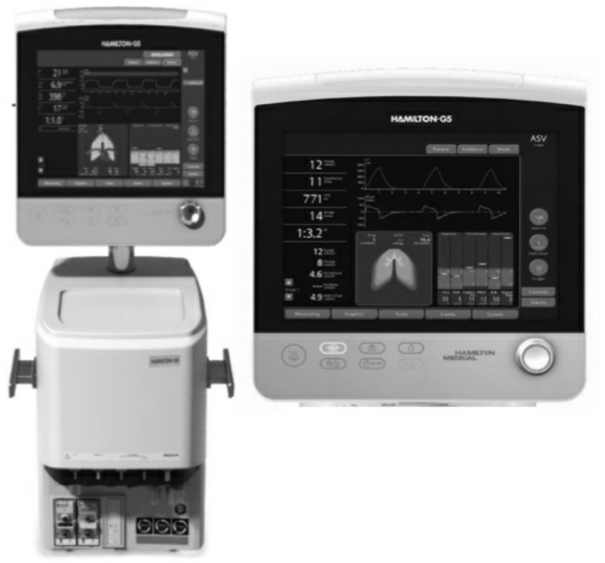

Hamilton G5

The Dräger Evita XL, Dräger Evita Infinity V500 and Dräger Savina 300 are pictured below.

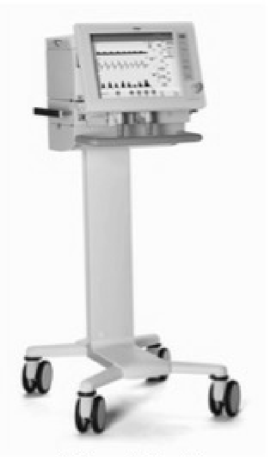
Dräger Evita XL

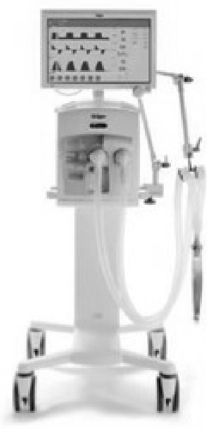
Dräger Evita V500

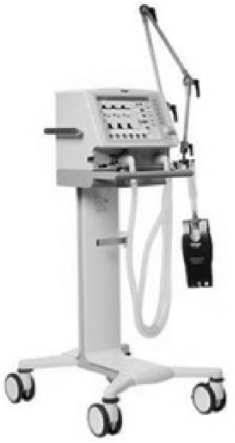
Dräger Savina 300

The Maquet SERVO-i and Maquet SERVO-u is pictured below.

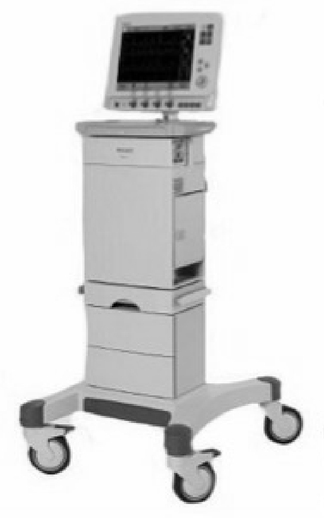
Maquet SERVO-i

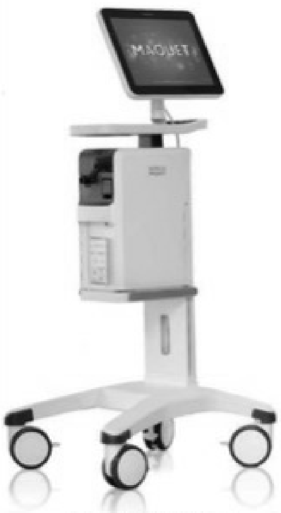

Maquet SERVO-u

* 12. How many years of experience do you have with the following devices (images pictured above)?
Example responses: n/a, <1, 2, etc.

Puritan Bennett 840:	
Puritan Bennett 980:	
Hamilton S1:	
Hamilton G5:	
Dräger Evita XL:	
Dräger Evita V500:	
Dräger Savina 300:	
Maquet SERVO-i:	
Maquet SERVO-u:	

* 13. When was the last time you used the following devices in your practice?

	No Experience	Currently using it	1-3 months ago	4-6 months ago	7-12 months ago	1-5 years ago	5-10 years ago	10+ years ago
Puritan Bennett 840:	○	○	○	○	○	○	○	○
Puritan Bennett 980:	○	○	○	○	○	○	○	○
Hamilton S1:	○	○	○	○	○	○	○	○
Hamilton G5:	○	○	○	○	○	○	○	○
Dräger Evita XL:	○	○	○	○	○	○	○	○
Dräger Evita V500:	○	○	○	○	○	○	○	○
Dräger Savina 300:	○	○	○	○	○	○	○	○
Maquet SERVO-i:	○	○	○	○	○	○	○	○
Maquet SERVO-u:	○	○	○	○	○	○	○	○

* 14. If you are currently using the following device(s) in your practice, how often do you use them?

	No Experience	Daily	Every 2-4 days	Once a week	Twice a week	Once a month	Less than once a month
Puritan Bennett 840:	○	○	○	○	○	○	○
Puritan Bennett 980:	○	○	○	○	○	○	○
Hamilton S1:	○	○	○	○	○	○	○
Hamilton G5:	○	○	○	○	○	○	○
Dräger Evita XL:	○	○	○	○	○	○	○
Dräger Evita V500:	○	○	○	○	○	○	○
Dräger Savina 300:	○	○	○	○	○	○	○
Maquet SERVO-i:	○	○	○	○	○	○	○
Maquet SERVO-u:	○	○	○	○	○	○	○
